# Congenital facial nerve palsy: Single center study

**DOI:** 10.3389/fped.2023.1077238

**Published:** 2023-02-20

**Authors:** Hermine Baelen, Anne-Marie Esschendal, Yannick De Brucker, Ina Foulon, Vedat Topsakal, Frans Gordts

**Affiliations:** ^1^ Department of Otorhinolaryngology, Head and Neck Surgery University Hospital UZ Brussel, Brussels, Belgium; ^2^ Faculteit Geneeskunde en Farmacie, Vrije Universiteit Brussel, Brussels, Belgium; ^3^ Department of Radiology, University Hospital UZ Brussel, Brussels, Belgium

**Keywords:** congenital facial nerve palsy, inborn facial nerve palsy, congenital syndrome, sensorineural hearing loss, imaging facial nerve

## Abstract

**Objectives:**

This study will list the most common comorbidities of congenital facial nerve palsy and how to detect and treat them, with special attention for ENT-problems such as hearing loss. Congenital facial nerve palsy is a very rare entity but in UZ Brussels hospital there was a follow-up of 16 children in the last 30 years.

**Methods:**

Literature review has been done, combined with thorough research of our own series of 16 children with congenital facial nerve palsy.

**Results:**

Congenital facial nerve palsy can be part of a known syndrome, most commonly Moebius syndrome, but can also appear solely. It appears often bilateral and with a severe gradation. In our series, hearing loss is frequently seen in association with congenital facial nerve palsy. Other abnormalities are dysfunction of the abducens nerve, ophthalmological problems, retro- or micrognathism and abnormalities of limbs or heart. The majority of the children in our series underwent radiological imaging (CT and/or MRI): the facial nerve but also the vestibulocochlear nerve and middle and inner ear can be evaluated.

**Conclusion:**

A multidisciplinary approach of congenital facial nerve palsy is recommended as it can affect various bodily functions. Radiological imaging needs to be done to acquire additional information that can be useful for diagnostic and therapeutic purposes. Although congenital facial nerve palsy may not be treatable itself, its comorbidities can be treated and improve the quality of life of the affected child.

## 1. Introduction

Facial nerve palsy is rare in children but very disturbing and frightening for parents. Its incidence in a pediatric population is estimated between 8% and 25% in literature (1). Congenital facial nerve palsies have a lower incidence of around 2 per 1,000 (2–8). They are attributed to either acquired etiologies such as perinatal traumata or to congenital developmental problems during embryonic or fetal life. It is generally accepted that perinatal traumata are the most common reason for acquired congenital FNP (facial nerve palsy) (3). These may arise when the mastoid tip is not yet fully developed at time of birth and the facial nerve is therefore covered only by soft tissue, making it vulnerable to compression trauma during birth (9). Developmental congenital FNP occurs often in combination with other inborn abnormalities. Sometimes a syndromic disease can be recognized, but isolated cases also occur. Unknown combinations of anomalies or rare syndromes may pose a challenge to identify but it seems evident that every newborn with FNP should be actively screened for other anomalies.

Two well-known syndromic causes of congenital FNP are Moebius syndrome and hemifacial microsomia. Moebius syndrome is diagnosed if the facial nerve and abducens nerve are both affected (10). Hemifacial microsomia means that there is asymmetry in the child's face, going from mild mandibular hypoplasia to facial nerve palsy combined with a deformed external ear (11). A child with hemifacial microsomia can have associated anomalies such as cardiac, renal or vertebral problems, for example with Goldenhar syndrome (12). There are still a lot of other possible causes of congenital FNP, such as VACTERL-syndrome, CHARGE-syndrome or isolated facial nerve agenesis.

Congenital FNP is not only a rare entity, but it is also not easy to differentiate between FNP (involving the orbicularis oris and orbicularis oculi muscles) and an isolated lower lip palsy. A meticulous clinical observation is imperative, and examining a child when it cries, can help to identify an isolated asymmetry at level of the mouth (13). An isolated lower lip palsy can be caused by hypoplasia of one of the lower lip muscles or by problems of the mandibular branch of the facial nerve (14, 15). Both clinical presentations may look very similar at a first glance but have absolutely different underlying pathophysiology and thus different clinical work-up.

The consequences of FNP may not be life threatening but are socially and emotionally devastating for patient and parents. In the worst case, it can even lead to social isolation. At least two reasons can be identified that may provoke a social burden. Firstly, the affected child can show less facial expression, possibly leading to social rejection or less social interaction. Secondly, when children become conscious of their handicap, of course it effects their psychological development. The physical handicap consists of difficulties with drinking or eating, continuous drooling (usually also socially disturbing) and ocular problems such as ulceration of the cornea when untreated (16–18). Altogether, the consequences of facial nerve palsy should not be underestimated. They influence a child's life and development in many ways.

In treatment strategies, there seems to be no golden standard on how to manage the condition. There is however a consensus to actively search and exclude other etiological causes that may be treatable (5). Surgical repair of facial palsy is widely debated and covers active and passive reconstructions of the facial nerve or its functions. Most studies in literature refer to adult-onset palsies, probably because pediatric cases are so scarce. There is no defined ideal age for surgery, let alone which procedure is preferable. Some surgeons state “the earlier the child is operated, the better recovery afterwards” but this approach is not substantiated with solid scientific evidence (19). Others propose surgery at age of 5 years (preschool age) to have some maturity of tissues but still be on time to reduce some social burden (2, 20). Most reports in literature are rather anecdotal and involve specific syndromes or surgical interventions and advocate a case-by-case management. This study aims to tackle this lack of knowledge. Meticulous delineation of this disorder and their individual management of probably the largest single center series may pave the way for future consensus. Based on a literature search on co-pathologies occuring with congenital FNP, we collected all clinically relevant data from patient charts of congenital FNP patients.

Here, we report all our cases of congenital FNP encountered over nearly 30 years in our clinic in order to characterize the trait. Secondly, we looked for correlations or even similarities in treatment or supportive management modalities to find more consensus in the management of congenital FNP.

## 2. Methods

**Figure 1:**
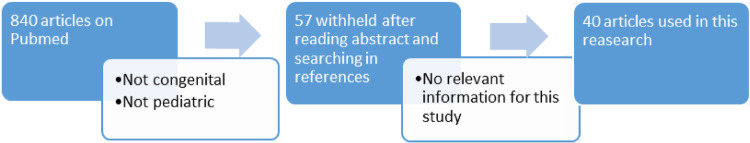
Literature search, article selection.

First, a literature search was performed on Pubmed to identify associated symptoms of congenital FNP. The search was limited to English literature that also provided an abstract. The following keywords were used: “congenital facial nerve palsy”, “asymmetric crying facies”, “Goldenhar syndrome and facial palsy”, “hemifacial macrosomia and facial palsy” and “CHARGE syndrome and facial palsy”. With these keywords, 840 publications were found on 03/03/2022. After reading the title, a first selection could already be excluded for our purpose. After reading the abstract and searching through footnotes, 57 articles were withheld for further reading (Figure 1). After thorough reading, not all articles had additional information on associated symptoms. In the end, 40 articles were identified that describe associated symptoms of congenital FNP.

Hereafter, we conducted a retrospective, single center study with approval of the medical ethical committee of UZ Brussel (B.U.N. 143201627518). The electronic medical records of all children with congenital FNP presenting at ENT-department of University Hospital Brussel (UZ Brussel) between 1992 and 2022 were screened. Moreover, an e-Health platform allowed even to consult electronic medical files from other hospitals in Belgium when parents gave informed consent. The e-Health platform is a nationwide network for hospitals in Belgium, where all actors of healthcare can exchange medical information, with permission and respect for the patient's privacy (21). In Belgium, patients have a free choice which doctor or specialist they consult. Accessing e-Health allowed collecting information on comorbidities that might have been treated outside our hospital.

The identified characteristics of congenital FNP from our literature were enumerated and analyzed with attention to all comorbidities in our population. Demographic data were studied. All available audiological tests and diagnostic imaging studies (MRI and/or CT) were also assessed. Specific CT-scans of the temporal bone were studied for anomalies using dedicated planning software (OTOPLAN®, CAScination AG, Bern, Switzerland) allowing to estimate cochlear duct lengths and even facial nerve anomalies. Although surgical treatment is beyond the scope of our study, also this information was gathered for our series and reported.

## 3. Results

The literature search and especially the 37 selected articles, show us which co-existing anomalies may appear with facial nerve palsy. Table 1 shows an overview of the results of this search. Hearing loss is reported by most other research groups. Problems with other cranial nerves or anomalies of the external, middle or inner ear are also frequently reported. Based on these studies, we defined subcategories to discuss our results.

**Table 1:** Results literature search.

	Total N	Hearing	External ear	Middle ear	Inner ear	Other cranial nerves	Cleft palate or uvula	Eyes	Cardiac	Limbs	Diagnosis
Ahn (29)	1		1					1	1		CHARGE
Grundfast (36)	2	1	2								Goldenhar ∑
Smith (9)	21		Multiple			5			3	1	Different diagnoses
Bergstrom (25)	35	12	14	5	3		9	2	3	2	Different diagnoses
Lin (3)	1	1			1						Unknown
Edwards (28)	24	16	Multiple	Multiple	Multiple			22	22		CHARGE ∑
Costa (7)	1	1									
Hamizan (37)	1	1	1		1	1					Unknown
Nicolai (38)	12	3									
Yetiser (8)	4					1					Unknown
Raveh (39)	2	2	2		2			2		1	Branchio-oculo-facial ∑
Sanchez (40)	1	1	1			1		1	1	1	CHARGE ∑
Carr (16)	29					15				3	Different diagnoses
Berker (30)	1	1						1	1		Goldenhar ∑
This study	16	8	7	8	7	9	2	10	6	6	

In the second part of our study, we identified twenty files of children with suspicion of congenital FNP at University Hospital Brussel between 1992 and 2022. In two patients, the diagnosis was modified to an isolated lower lip palsy in the follow-up. In two other children, an association with infectious middle ear problems was suspected to be a provocative factor for the facial palsy. Those four children were excluded from further analyses. In total we have studied a cohort of 16 children with congenital facial nerve palsy. Thirteen patients or their parents gave permission to check their e-Health platform.

### 3.1. Demographics and etiology

The age at time of diagnosis ranges between 1 day and 6 years. 75% of the children had their diagnosis before the age of 1 year (Table 2).

**Table 2:** Demographics and etiology.

Patient-number	Age (in years)	Sex	Diagnosis	Age at diagnosis (months)	Side	House-Brackmann
1	34	M	CHARGE ∑	Directly after birth	Left	IV or more
2	28	M	Goldenhar ∑	1	Left	IV
3	21	F	Trisomy 18, mosaicism	36	Left	IV or more
4	12	M	Branchio-oto-renal ∑	10	Right	III
5	18	F	Thick and irregular FN	6 years	Right	IV
6	13	F	Agenesis FN	22	Right	IV
7	10	M	Poland-Moebius ∑	5	Bilateral	IV or more
8	10	M	Hypoplasia 2nd part FN	11	Left	IV
9		M	Trisomy 18	Directly after birth	Left	-
10	8	F	Poland-Moebius ∑	4	Bilateral	IV or more
11	9	M	Poland-Moebius ∑	18	Left	IV
12	6	M	Agenesis FN	7	Left	IV or more
13	4	M	Moebius ∑	10	Left	IV or more
14	11	M	Goldenhar ∑	3	Left	Not clear
15	7	F	VACTERL ∑	Directly at birth	Right	III
16	1	F	Moebius ∑	1	Bilateral	IV

∑, syndrome, FN, facial nerve.

There are ten boys (62.5%) and six girls (37.5%) included in this heterogenous study population. Three children have a bilateral FNP (18.75%) and thirteen a unilateral palsy; nine a palsy on the left side (56.25%) and four on the right side (25%).

Children with congenital FNP seem to have a severe form of facial nerve palsy. 75% of the children in our study have a House-Brackmann gradation IV or more. The average House-Brackman grade is 3.8 with a median grade of 4. The cases with bilateral palsy were graded on average House-Brackman grade 4 with no clear difference between left and right side.

One child, diagnosed with trisomy 18 and characterized with congenital FNP on the left side, deceased a few months after birth (number 9).

Four patients (25%) underwent surgical intervention at young age, all under the age of 10 years. An example of such an operation is a cross-face graft with the suralis nerve and a transfer of the gracilis muscle. Parents of four others also consulted for surgical correction, but possible interventions were postponed to older age.

The etiology of congenital FNP in our series is rather heterogenic. We found eight different causes, listed in Table 2. The most common cause is Moebius syndrome. It appears in five children with congenital FNP in our study (31.25%), among whom those three with bilateral palsy. All five children have also a palsy of the abducens nerve, a typical symptom of Moebius syndrome. In 60% of those Moebius cases, it is combined with Poland sequence which is defined as congenital absence of the pectoral head with ipsilateral hand deformity (22).

In 25% of the cases, the clinical findings could not result in a diagnosis, so it was necessary to do radiological imaging. Performing an MRI scan resulted in an additional diagnosis of agenesis of the facial nerve, hypoplasia of the second part of the facial nerve or a thickened and irregular facial nerve in four children (number 5, 6, 8 and 12).

### 3.2. Ear and hearing

50% of the children (8 patients) have some degree of hearing loss. All types of hearing loss were seen, even separate types of hearing loss co-occurred in one patient (number 15, conductive hearing loss on the left side and sensorineural loss on the right side). Two of those eight children have unilateral hearing loss, six bilateral (Table 3). The children with bilateral hearing loss surprisingly have only a unilateral facial nerve palsy. If we classify the severeness of the hearing impairment, four children have a profound hearing loss (three sensorineural, one conductive), one has a severe hearing loss (sensorineural), one a moderate hearing loss (conductive) and two have a slight impairment (conductive) (WHO's Grades of hearing impairment). The five children with Moebius syndrome all have normal hearing thresholds.

**Table 3:** Results hearing test.

Patient-number	Side FNP	Side hearing loss	Hearing threshold (fletcher if pure tone audiometry)		Type of hearing loss	Hearing evaluation	Management hearing loss
			Right	Left			
1	Left	Bilateral	23 dBHL	23 dBHL	Sensorineural ipsilateral and conductive contralateral	Pure tone audiometry	Hearing aid broke, didn't buy a new one
2	Left	Bilateral	60 dBnHL	60 dBnHL	Conductive	BERA	BAHA
3	Left	None				Pure tone audiometry	
4	Right	Bilateral	23 dBHL	27 dBHL	Conductive	Pure tone audiometry	No hearing aid on the right side anymore
5	Right	Ipsilateral	>120 dBHL		Sensorineural	Pure tone audiometry	
6	Right	None				AABR + ASSR	
7	Bilateral	None				BERA	
8	Left	None				AABR + ASSR	
9	Left	Bilateral	80 dBnHL	80 dBnHL	Sensorineural	BERA	
10	Bilateral	None				AABR + ASSR DPOAE's	
11	Left	None presumed			Presumably no hearing loss	No testing found	
12	Left	Bilateral	>95 dBnHL	>95 dBnHL	Sensorineural	BERA	Brain stem implant
13	Left	None				BERA	
14	Left	Ipsilateral		>120 dBHL with air-conduction, normal bone-conduction	Conductive	Pure tone audiometry	BAHA
15	Right	Bilateral (ipsilateral worse)	>95 dBnHL	50 of 60 dBHL	Sensorineural ipsilateral and conductive contralateral	BERA, pure tone audiometry at age of 3 months	BAHA softband, bilateral
16	Bilateral	None				AABR + ASSR	

There were several hearing tests used, depending on the cooperativeness of the child. Four children (25%) underwent pure tone audiometry, seven (43.75%) brainstem evoked response audiometry (BERA) and four (25%) automated auditory brainstem response (AABR, used in Flanders as screening of newborns). For one child we could not retrieve hearing evaluation also not in files of other centers for which parents gave consent.

Six of the eight patients with hearing loss were fitted with hearing improving aids or implants: two have a behind-the-ear hearing aid, three have a bone anchored hearing aid (BAHA) and one patient has a brain stem implant. One patient with hearing loss is deceased (number 9). The last patient (number 5) with hearing loss, only unilateral, was lost to follow up. The compliance of the patients with a behind-the-ear hearing aid is not good, both do not wear it anymore.

We also observed anatomical deformations of the auricle and the external auditory canal (Table 4). Seven patients have deformations of the pinna, varying from mild deformations such as cup-shaped ears, up to anotia. The Weerda classification is used for the different types of microtia in Table 4 (23). In three patients (18.7%) an associated atresia of the external auditory canal is observed on the ipsilateral side. We observe a strong correlation between the presence of atresia of the external auditory canal and ossicular chain anomalies in our series of congenital FNP. These anomalies can vary between fusion of malleus and incus, up to the absence of the complete ossicular chain. In this study, we notice that the atresia is always, in all three cases, combined with a problem of the ossicular chain (number 2, 14, 15).

**Table 4:** Deformations outer, middle and inner ear.

Patient-number	External ear	Middle ear	Inner ear	
			Cochlea	Vestibular system
1	Normal	Often otitis media with effusion	Normal on CT and MRI	Bilateral absence of semicircular canals on CT
2	Bilateral microtia (Weerda grade 2); left pre-auricular tags	Bilateral atresia external auditory canal, left side no ossicular chain, right side abnormal chain	Normal on CT	Normal on CT
3	Normal	Often otitis media with effusion, transtympanic tubes	Normal on CT	Normal on CT
4	Cup-shaped ears (Weerda grade 1), pre-auricular pits bilateral	Often otitis media with effusion, transtympanic tubes, bilateral fusion between malleus and incus, possibly no contact with stapes	Hypoplasia of apical and second winding	Interruption posterior semicircular canal right side, abnormal morphology lateral semicircular canal bilateral, plump vestibulum left side
5	Normal	Often otitis media with effusion, transtympanic tubes, small antrum right side	Mondini malformation	Absent semicircular canals, abnormal morphology vestibulum
6	Low implanted ears (limit)	Normal	Normal	Bilateral dehiscence SSCC
7	Low implanted ears	Normal	Normal	Right side dehiscence SSCC
8	Normal	Normal	Normal	Right side possible dehiscence superior and posterior SCC
9	Low implanted left ear	No information	No information	No information
10	Normal	No information	Presumably normal because normal hearing	No information
11	Normal	No information	No information	No information
12	Normal	Often otitis media with effusion	Normal	Normal
13	Normal	normal	Normal on MRI	Normal on MRI
14	Skin tags right side, abnormal morphology left side (Weerda grade 1)	Left side atresia externa auditory canal, fusion incus and malleus, otitis media with effusion	Normal on MRI	Normal on MRI
15	Cauliflower ear right side (Weerda grade 3), hypoplasia superior part left side (Weerda grade 2)	Atresia external auditory canal bilateral, problems with ossicular chain	Normal on CT and MRI	Normal on CT and MRI
16	Normal	No information	No information	No information

### 3.3. Imaging

**Figure 2:**
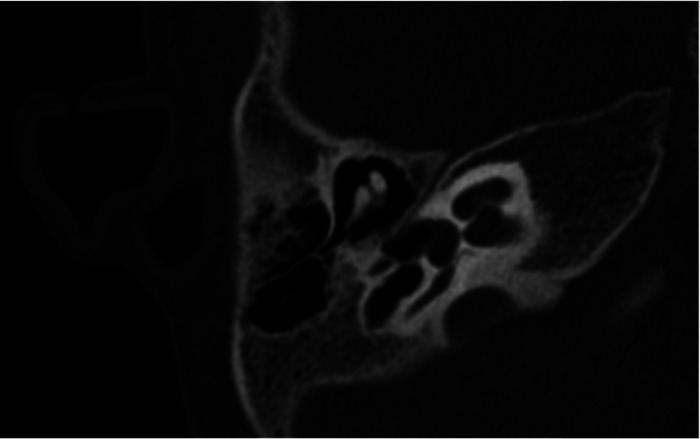
CT scan of a narrow fallopian canal of the facial nerve.

In ten patients, a computed tomography of the temporal bone was performed, requiring general anesthesia in eight cases. In six patients (37.5%), a narrow fallopian canal of the facial nerve is noticed. An example of this can be seen in Figure 2. Abnormalities are seen in all three segments of the facial nerve, consisting of the labyrinthine segment, the mastoid segment and the horizontal segment. The abnormalities can be a combination of a problem in two or three segments at the same time in our study (Table 5).

**Table 5:** Results CT scan.

Patient-number	CT scan		
	Labyrinthine	Mastoid	Horizontal
2	Normal	Not clearly seen on the left side, right side normal	Not clearly seen on the left side, right side normal
3	Normal	Normal	Abnormal course of narrowed third portion
4	Normal	Normal	Normal
5	Broadened	Normal	Normal
6	Narrow canal	Narrow	Narrow
7	Right side normal, left side a bit hypoplastic	Bilateral hypoplastic, left side more than right	Bilateral normal
8	Normal	Narrow right side, very narrow left side	Normal right side, very narrow left side and then becoming normal
14	Normal	Normal	More anterior than normal
15	Absent right side	Absent right side	Absent right side

No CT-scan was performed in patient 1, 9, 10, 11, 12, 13, 16.

Fourteen patients in our series of sixteen underwent an MRI of the temporal bone, thirteen under general anesthesia. In six cases, a complete agenesis of the facial nerve on the side with the facial nerve palsy could be diagnosed. In three other patients, a severe hypoplasia was observed and only some fibers were suspected to represent the facial nerve. In one patient (number 5), the facial nerve is thickened and irregular.

It is noteworthy that there are three patients in our study (number 7, 12 and 13) with a bilateral agenesis or hypoplasia of the facial nerve, according to the protocol of the radiologist, but a unilateral palsy. In two children with facial nerve anomalies on MRI, this MRI showed also uni- or bilateral absence of the vestibulocochlear nerve (number 5 and 12).

On CT-scan, there were also abnormalities found in the inner ear. There are more abnormalities found in the vestibular system than in the cochlea (six patients versus two patients). In the vestibular system there can be an absence of the semi-circular canals (two patients), a dehiscence of the superior semi-circular canal (three patients) or an abnormal morphology (one patient). On cochlear level there is an incomplete partition type II and a cochlear hypoplasia with hypoplastic middle and apical turns (number 5 and 4) (24).

The CT scans of six patients have been uploaded into Otoplan for additional calculations. The cochlear duct length of four patients with normal morphology of the cochlea varies between 28 and 36 mm. Two patients have a deformation of the cochlea (number 4 and 5). Their cochlear duct length is 22.8, 31, and 35 mm.

### 3.4. Associated anomalies

Only two children have a unilateral isolated congenital FNP, due to facial nerve agenesis diagnosed on MRI in our series (number 6 and 8). Fourteen patients (87.5%) have associated traits that could have been substantiated. An overview of these associated anomalies is shown in Table 6.

**Table 6:** Associated anomalies.

Patient-number	Additional cranial nerve problems	Eyes	Face	Mouth	Cardiac
1		Coloboma bilateral	Choanal atresia	Ogival palate, bifid uvula	AV-nodal re-entry tachycardia
2		Left side obstruction nasolacrimal canal and lagophtalmos	Hemifacial microsomia, micrognathism, no TMJ left side	Glossoptosis, bifid uvula	ASD (II)
3				Abnormal dental position, ogival palate	
4	Problem palatal branch of nervus X		Retrognathism	Palsy right side palatum	
5	Absent N. vestibulocochlearis right side	Prominent eyeballs			Mitral valve prolapse with very mild mitral valve insufficiency
6					
7	Bilateral paresis N. abducens	Strabismus	Pierre-Robin		
8					
9		Exoftalmia left side	Retrognathism		
10	Bilateral paresis N. abducens	Strabismus	Retrognathism		
11	Bilateral paresis N. abucens	Strabismus, esotropia left side			
12	Absent N. vestibulocochlearis bilateral	-	Plagiocephalia		Small foramen ovale, hemodynamically irrelevant
13	Bilateral paresis N. abducens	Ptosis bilateral (R>L), blepharophimosis, epicanthus inversus syndrome	Plagiocephalia		
14		Amblyopia right side because of esotropia and anisometropia, coloboma left eyelid	Hypoplasia mandibula, plagiocephalia		Tricuspid valve insufficiency
15	Absent N. (vestibulo)cochlearis right side		Possibly choanal atresia		
16	Bilateral paresis N. abducens	Ectropion left side, papillary coloboma	Limited retrognathism		Pulmonary valve stenosis

Patient-number	Limbs	Kidneys	Genitals	Thorax	Brain	Other
1			Pituitary hypogonadism			
2						Tracheotomy, gastrostomy
3	Hand dysplasia, unique palmar fold on the right side				She functions as if there is a frontal injury	
4	Joint hypermobility syndrome, left foot in valgus	Hydronephrosis left side	Adhesions			Bilateral neck fistulas
5						
6						
7	Fingerlike thumbs, syndactyly left side			Asymmetric thorax, hypo- or aplasia of left M. pectoralis		Gastrostomy
8						
9	Camptodactily left side, long fingers bilateral, club feet bilateral	Hydronephrosis right side	Hypospadias	Asymmetric thorax		Vocal cord paralysis with obstruction of airway
10	Brachysyndactyly left side 2-5, syndactyly right side 1-2					
11	Arm and hand right side smaller then left side, syndactyly 2-3 right side			Hypoplasia right M. pectoralis		
12						Mongolion spot sacral and lumbal
13						Laryngomalacia
14				Scoliosis D1-D6, cervical rib	Autism, meningitis	Hypogammaglobulinemia with IgG2 deficiency
15		Neurogenic bladder		Scoliosis, kyphosis	Psychomotor retardation	Esophageal atresia, trache-oesophageal fistula, anal imperforation, cloaca, short stature
16						

#### 3.4.1. Craniofacial abnormalities

The most frequently affected cranial nerves, besides the facial nerve, are the abducens nerve (VI) and the vestibulocochlear nerve (VIII). Only the children with Moebius syndrome have clinical and radiological deformation of the abducens nerve in our study. Two children (number 5 and 12) have an absent vestibulocochlear nerve, one unilateral and one bilateral. Those ears are deaf ears.

Ophthalmological examinations report an anomaly in 56.25% of the children. Coloboma and strabismus are seen respectively in two and three cases.

Retrognathism and micrognathism are also frequently seen in our series, namely with six children. It is combined with syndromes such as Goldenhar syndrome, branchio-oto-renal syndrome, Moebius syndrome or trisomy 18. Other anomalies that are seen with most of these syndromes are an ogival palate or a bifid uvula. There were no children with a cleft palate in this study. Two have a bifid uvula (number 1 and 2). Choanal atresia is described with CHARGE syndrome and was present in the patient in our study with that syndrome.

#### 3.4.2. Limb abnormalities

In total there are six children (37.5%) with limb deformations. Problems of the upper limbs, more specifically the hands, are more common than those of the lower limbs. Five children (31.2%) have syndactyly or other hand deformations (number 3, 7, 9, 10 and 11).

#### 3.4.3. Cardiac abnormalities

In our study, different categories of cardiac anomalies are seen. There are valve problems, anatomical deformations and electrophysiological problems. In total, there are six children (37.5%) with a cardiac anomaly. Three have a valve problem. The mitral, tricuspid or pulmonary valve can be involved. Two children have an atrial septal defect or a small oval foramen. The last child has an AV-nodal re-entry tachycardia.

#### 3.4.4. Other abnormalities

In this study we saw also anomalies of the genitals, the kidneys, the brain and the thorax, and in addition also some rare interventions were needed such as a gastrostomy or a tracheostomy. An overview of the different problems can be found in Table 6. Some important ones are hydronephrosis, Chiari malformation, scoliosis or kyphosis and neck fistulas. Neck fistulas are typically seen in branchio-oto-renal syndrome.

## 4. Discussion

Although congenital FNP is a very rare condition, we have managed treatment of 16 cases over 30 years in our tertiary referral center, UZ Brussels with a specific children hospital. Generally, we noticed a severe grade of facial palsy, with 75% of the children with a House-Brackmann gradation of IV or more which is consistent with current literature (3). In our population, the palsy was predominantly unilateral, although there are researchers who claim that a congenital FNP is usually bilateral (25). Some assume that the difference between traumatic or congenital FNP can be made based on whether the paresis is uni- or bilateral. Our results here suggest that a unilateral FNP is not always caused by birth traumata and a single sided congenital FNP can occur.

Although it can occur solitarily, our series suggests that congenital FNP is not a condition of disease on its own but has rather very heterogenic etiologies. We had eight separate diagnoses that encomprized congenital FNP in sixteen cases. Other studies also noticed that there can be a great variety of causes (9, 17, 26). The obvious and most common diagnosis that comes to mind in neonatal FNP is probably Moebius syndrome. This condition is the textbook example, but most residents will require some years in pediatric ENT before they encounter their first case. Our series describes 16 cases with congenital FNP in about 30 years, which means that this is not even an annually diagnosed condition.

Nevertheless, a systematic approach in the work up will also reveal other etiologies or co-occurrence of other conditions such as the associated symptoms of Goldenhar syndrome, branchio-oto-renal syndrome, CHARGE syndrome and VACTERL syndrome. Children with CHARGE syndrome often have a facial palsy (27). It is not yet clear in those patients if this is caused by a central lesion at the level of the brainstem or if it is caused by a problem in the middle ear (28, 29). For those other syndromes, it is rather rare that they are combined with facial palsy (30, 31).

The contributions of the clinical history taking and clinical examination are important to be able to diagnose a syndromic cause. The importance is not (only) missing a diagnosis of a syndrome but much more the importance of missing a possibly co-existing treatable trait. If there are other anomalies seen, this points rather in the direction of a syndromic cause. If there are no other obvious defects, the differentiation between a traumatic facial palsy or congenital FNP may be difficult (8). But even with a history of birth trauma, a complete work-up is recommended to exclude other causes and to avoid therapeutic implications (5). It seems evident to perform imaging if other anomalies, and especially neurological abnormalities, are seen (26). However, even in our series we have encountered cases without imaging. Both CT and MRI are indicated, because a normal facial canal on CT does not exclude the presence of a hypoplastic facial nerve, mostly better seen on MRI (32). Of course, consideration about whether general anesthesia is necessary or what amount of x-ray exposure on CT scans is acceptable for children, and the logistic problem of transporting a child from MRI to CT under anesthesia are all different for each hospital. Sometimes, some of these factors may delay or even cancel the required imaging for congenital FNP. If it is unclear where the pathology is situated, MRI with intravenous gadolinium contrast is the modality of choice. Ultrasound can be useful for assessing pathology within the parotid gland, although this is not often performed (33). Diagnostic examinations should be performed as soon as possible or as soon as the general health of the baby allows it. If the examinations take place a few weeks or months after birth and there is still a facial palsy existing, then this suggests rather an underlying developmental cause than a traumatic one (34). It is important to be able to make this distinction, both for diagnostic and therapeutic management as also for perhaps medicolegal aspects (1, 27). Faster CT scans (and cone beam CT scans) will allow lesser x-ray exposure and faster scanning that may not even require anesthesia. In our series eight of ten CT scans and thirteen from fourteen MRI scans required general anesthesia to keep the child immobile for image quality reasons. It is not even logistically possible in our facilities to perform both imaging modalities within the same anesthesia since both devices are in separate buildings. For those reasons, many discussions with pediatric anesthetists result in a second-best solution which is opting for MRI or CT and not performing both. In terms of diagnostic yield, both imaging modalities are complementary and cannot replace each other.

There were eight children with an abnormality at the level of the facial canal seen on CT. Six of them (37.5%) have a narrowed canal. This is comparable with the results of Terzis et al. where thirteen of 41 children (31.7%) have a narrowed canal (32). Sometimes, central lesions can be seen on imaging, especially on MRI, for example an abnormal nucleus (4). Jemec et al. state that congenital FNP can not only be caused by peripheral damage to the facial nerve or syndromes but also by central nerve system-abnormalities (4). On MRI, the diameter of the facial nerve can be well seen. It can be compared with the other side to tell if there is a hypoplasia. In our study, two children have bilaterally an abnormal facial nerve but just a unilateral palsy. Other investigators confirm this finding that a malformation of a cranial nerve can be asymptomatic (6, 7).

The fact that we have one child in our series in which we could not retrieve any audiological work-up is worrying if we see that in the others about 50% have some sort of hearing impairment. Again, the goal is not to determine features of a syndromic disease or a genetic trait but much more to look for treatable or manageable anomalies that are associated with congenital FNP. Although no obvious link between congenital FNP and SNHL or cochlear anomalies may come to mind immediately (35), the correlation is present in a subset of our data, the children who do not have Moebius syndrome, and more often reported in literature (9). A possible explanation for this link is that the separation of the facial nerve and vestibulocochlear nerve takes place at week 5 or 6 of pregnancy (27). So, a problem *in utero* before this time can affect both nerves. There are different hypotheses known that explain why there can be a problem at level of the facial nerve early in embryonic life. The hypothesis that comes back most often is the one that states that there is an interruption of embryonic blood supply due to a problem of the subclavian artery (8, 22).

**Figure 3:**
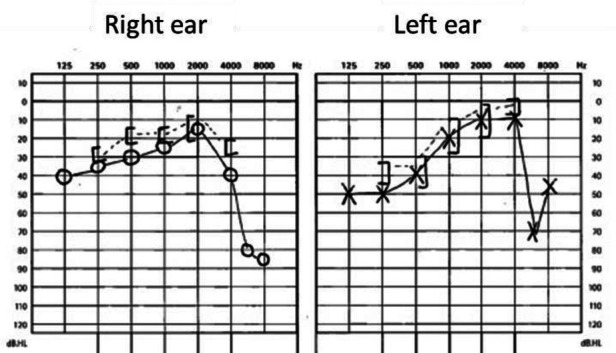
Hearing test of patient number 1.

It is therefore mandatory to evaluate hearing in every case with congenital FNP according to the state-of-the-art investigations at an as early age as possible to avoid language and speech developmental delay. We confirm this statement as in our study 50% of the children have some degree of hearing loss. The hearing test should be adjusted to the patient's age, for example BERA for a baby and pure tone audiometry for children aged 4 years or older. Hearing loss can be severe with the need of a behind-the-ear hearing aid or even surgical solutions such as bone anchored hearing aid or a brainstem implant. Interestingly, in CHARGE-syndrome facial palsy is a predictor of ipsilateral neurosensorial hearing loss (28). Our patient with CHARGE syndrome has a neurosensorial low- and high frequency hearing loss on the ipsilateral side and a mixed hearing loss on the contralateral side, with the neurosensorial part mostly in the high frequencies (Figure 3). As in a normal setting, every audiological examination starts with a clinical examination including otoscopy and examination of the external ear.

Smith et al. reported that external auditory canal atresia and some degree of microtia are often associated with congenital FNP (9). In our study there are also multiple children with those problems. It is not yet clear how it comes that the outer ear and the external auditory canal are also affected in children with congenital FNP. Presumably it is caused by the syndromic features of the disease.

Other otological abnormalities, for example of the middle or inner ear, can be seen with CT and MRI. In our study, we saw several children with problems of the vestibular system seen on imaging. It is not known if they have vertigo because it was not systematically asked in the anamnesis and the children did not undergo c-VEMP or another vestibular test.

There are remarkably few children with an abnormality of the cochlea seen in our series. One child has an incomplete partition type II with associated sensorineural hearing loss and another child has cochlear hypoplasia with hypoplastic middle and apical turns with a small conductive hearing loss due to atresia of the external auditory canal. So, although there are three children with deafness due to sensorineural hearing loss, there is only one child of those three with an anomaly on cochlear level seen on imaging. Nevertheless, imaging stays important, not only to have a better look on the facial nerve but also to see if there are vestibular or cochlear malformations. Besides, CT scan is important to evaluate the middle ear, especially when there is a deformation of the auricle and the external auditory canal.

Bergstrom et al., stated that cleft deformations are frequently seen in children with congenital FNP, namely in eight of 35 children (22.8%) (25). In our study, only two children of sixteen (12.5%) have a bifid uvula and not one child has a lip or palatal cleft. This proves again that a thorough examination of the newborn must be performed. Another part of this thorough clinical examination is the testing of other cranial nerves (27). Attention must be paid to swallowing and nutrition. Syndromic children may also have difficulties with swallowing (27). This can be caused by an afunctional glossopharyngeal nerve. Additionally, children should be referred to an ophthalmologist to check if vision is normal and if eye movements are within normal range.

## 5. Conclusion

Management of congenital FNP is multidisciplinary and starts with the most basic methodology in medicine: a thorough history taking with the parents, full clinical examination including otoscopy and with attention for deformations of the head, the limbs, the thorax and auscultation of the heart. An appropriate evaluation of hearing and vision acuity and radiological imaging are imperative. Hearing tests need to be performed as early as possible whereas there can be debate about the timing and modality of imaging techniques. Usually, all children benefit from CT and MRI imaging. Radiologic imaging is an important part of the diagnostic investigations as an abnormality on the peripheral course of the facial nerve can be seen. Additionally, the middle and inner ear can be evaluated.

Congenital FNP may not be immediately treatable, most children with facial nerve palsy have additional problems that may be treatable. It is important to know which other abnormalities can appear in combination with congenital FNP to give the best medical support to those children and their parents. Referral to a specialized, multidisciplinary center including geneticists seems mandatory as well, although the trait may not be life threatening.

## Data Availability

The original contributions presented in the study are included in the article/Supplementary Material, further inquiries can be directed to the corresponding author.
